# Effect of the Chemical Composition of Free-Terpene Hydrocarbons Essential Oils on Antifungal Activity

**DOI:** 10.3390/molecules24193532

**Published:** 2019-09-29

**Authors:** Ghada Ben Salha, René Herrera Díaz, Olfa Lengliz, Manef Abderrabba, Jalel Labidi

**Affiliations:** 1 Chemical and Environmental Engineering Department, University of the Basque Country UPV/EHU, Plaza Europa, 1, 20018 Donostia-San Sebastián, Spain; bensalhaghadaangel@gmail.com (G.B.S.); reneherdiaz@gmail.com (R.H.D.); 2 Laboratory Materials, Molecules and Application, Preparatory Institute for Scientific and Technical Studies, Marsa 2070, Tunisia; olfalenglizridene@gmail.com (O.L.); abderrabbamanef@gmail.com (M.A.); 3 Faculty of Sciences of Tunisia, University of Tunisia El Manar, Farhat Hached University Campus PB 94-Rommana, Tunis 1068, Tunisia; 4 University of Carthage, National Institute of Applied Science and Technology, BP 676, CEDEX, Tunis 1080, Tunisia

**Keywords:** *Carum carvi* L., *Origanum majorana* L., (−)-carvone, terpin-4-ol, antifungal activity, free-terpenes hydrocarbons

## Abstract

In this study, *Carum carvi* L. essential oil (CEO) and *Origanum majorana* L. essential oil (MEO) was steam-distillated under reduced pressure. We henceforth obtained three fractions for each essential oil: CF_1_, CF_2_, CF_3_, MF_1_, MF_2_, and MF_3_. Then, these fractions were characterized using the gas chromatography–mass spectrometry (GC-MS) technique. The results indicated that some fractions were rich in oxygenated compounds (i.e., CF_2_, CF_3_, MF_2,_ and MF_3_) with concentrations ranging from 79.21% to 98.56%. Therefore, the influence of the chemical composition of the essential oils on their antifungal activity was studied. For this purpose, three food spoilage fungi were isolated, identified, and inoculated in vitro, in order to measure the antifungal activity of CEO, MEO, and their fractions. The results showed that stronger fungi growth inhibitions (FGI) (above 95%) were found in fractions with higher percentages of oxygenated compounds, especially with (−)-carvone and terpin-4-ol as the major components. Firstly, this work reveals that the free-terpenes hydrocarbons fractions obtained from MEO present higher antifungal activity than the raw essential oil against two families of fungi. Then, it suggests that the isolation of (−)-carvone (97.15 ± 5.97%) from CEO via vacuum distillation can be employed successfully to improve antifungal activity by killing fungi (FGI = 100%). This study highlights that separation under reduced pressure is a simple green method to obtain fractions or to isolate compounds with higher biological activity useful for pharmaceutical products or natural additives in formulations.

## 1. Introduction

Over the last decade, several studies have shown the potential of using essential oils (EOs) as complementary medicine, i.e., for fungal diseases treatments. It is worth noting that most of these substances were declared as alternatives to synthetic fungicides from a natural origin [1,2,3]. Several studies identified EOs from carum (*Carum carvi* L.) (CEO) and origanum (*Origanum majorana* L.) (MEO) as potential antifungal agents against molds and food spoilage fungi [4,5,6]. However, there are not enough studies that clearly describe the effect of different molecules found in EOs and which could be effective as antifungal agents. Generally, these natural products are complex mixtures of terpenes (terpenes hydrocarbons and oxygenated components) with a variable degree of lipophilic and hydrophilic compounds [7]. It has been proven that the physical nature of any oxygenated compound favors the penetration to the fungi cell membrane, which increases the antifungal effect [8,9]. However, hydrocarbons compounds seem to have a limited contribution to the antifungal activity, as is mentioned in the literature [10,11]. In view of these considerations, the deterpenation process is an effective choice to increase the yields of oxygenated terpenes and to remove hydrocarbons (terpeneless EOs), which enhances the quality of EOs with regards to its biological activity. Moreover, this could promote their commercial value and even step up the possibility of upscaling the process [12].

The aim of this work was to study the chemical profile of *Carum carvi* L. and *Origanum majorana* L. EOs and their fractions were obtained with vacuum distillation. Further, we investigated relations between free-terpenes hydrocarbons fractions and major components and their antifungal effect. The main focus was to examine the inhibitory effects of these essential oils before and after the deterpenation process in order to identify the responsible terpenes of antifungal activity against Rizopus (*R. oryzae* and *R. stolonifier*) and *Aspergillus* (*A. penicillioides*) fungal strains. To the best of our knowledge, this is the first study that provides terpenless caraway essential oil with high purity of (−)-carvone 97.15 ± 5.97% using the under reduced pressure steam distillation method.

## 2. Results

### 2.1. Chemical Composition of EOs and Their Fractions

The results from the GC-MS analyses are reported in Table 1. The identification was established using the retention time lock (RTL) and the mass-to-charge ratio (*m/z*) of the largest peaks. The compounds quantification was based on the normalized peak area without using correction factors. The chemical results of MEO and its fractions were a part of our previous published work [10], which demonstrates that reduced pressure steam distillation was an effective method used to obtain deterpenated fractions from *Origanum majorana* L. essential oil. The interesting results of this simple green technique allowed us to test it on *Carum carvi* L., then to compare between MEO and CEO chemical compositions.

The main components of the EOs were monoterpenes, while sesquiterpenes and other compounds were found in a small amount. However, there were similarities only within each EO and its fractions but not similarities between the dominant components of MEO and CEO.

Caraway essential oil (CEO) was dominated by oxygenated monoterpenes (77.43%) followed by monoterpene hydrocarbons (22.48%) without any trace of sesquiterpenes. Within the oxygenated monoterpenes, (−)-carvone (74.25%) was the main compound. Among the monoterpene hydrocarbons, D-limonene (22.22%) was dominant.

On the other hand, the main compounds found in the marjoram essential oil (MEO) were terpin-4-ol (27.32%), γ-terpinene (15.73%), and α-terpinene (11.08%). This EO contained a complex mixture of oxygenated monoterpenes (47.36%), monoterpene hydrocarbons (50.70%), and a small amount of sesquiterpenes hydrocarbons (4.94%).

Regarding the CEO fractions, nine compounds were identified in the first fraction (CF_1_), in which D-limonene (77.35%), (−)-carvone (20.18%), and β-myrcene (1.54%) were the major components. Moreover, the composition of the second fraction (CF_2_) displayed the same chemotype as the CEO but with slight proportional differences. The third fraction (CF_3_) was composed of 13 constituents but was mainly dominated by (−)-carvone (97.15%).

In the case of the MEO fractions, fifteen compounds were identified in the first fraction (MF_1_), whereas γ-terpinene (27.53%), α-terpinene (21.15%), and 4-(10)-thujene (11.17%) represented the major components. In the second fraction (MF_2_), 17 constituents were identified with terpinen-4-ol (54.39%), *cis*-sabinene hydrate (10.00%), and β-fenchyl alcohol (9.63%) as the main components. Finally, 21compounds were found on the third fraction (MF_3_), mainly represented by terpinen-4-ol (48.60%), *cis*-β-fenchyl alcohol (23.84%), and *cis*-sabinene hydrate (5.79%).

### 2.2. Antifungal Effect of EOs and Fractions

**Figure 1:**
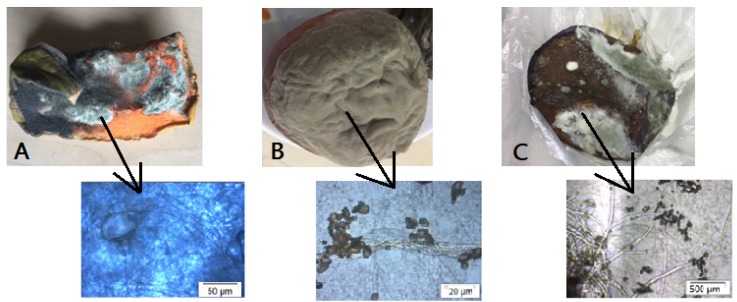
Spoilage fungi: (**A**) *R. oryzae* from pumpkins, (**B**) *R. stolonifier* from peaches, and (**C**) *A. penicillioides* form prunes.

All fruit spoilage fungi were isolated using the agar plate method (APM) and identified as follows: *Rhizopus oryzae* from pumpkins, *Rhizopus stolonifier* from peaches, and *Aspergillus penicillioides* from prunes (Figure 1). The identification was based on their growth and morphological characteristics.

**Figure 2:**
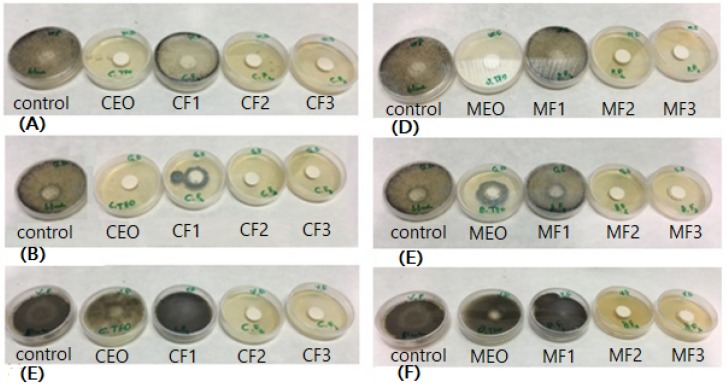
Visual observation of fungal growth inhibition (FGI) of *Carum carvi* L. essential oil (CEO) and its fractions against (**A**) *R. oryzae*, (**B**) *R. stolonifier*, and (**C**) *A. penicillioides*. FGI of *Origanum majorana* L. essential oil (MEO) and its fractions against (**D**) *R. oryzae*, (**E**) *R. stolonifier*, and (**F**) *A. penicillioides*. Media after seven days at 27 ± 1.5 °C.

After seven days of fungal exposition, the percentage of growth inhibition (FGI %) was considerable in the CEO and its fractions CF_2_ and CF_3_, in addition to the MEO and its fractions MF_2_ and MF_3._ The values are listed in Table 2; a visual appearance is shown in Figure 2.

The values of inhibition were more than 50% when both *Rhizopus* strains (*oryzae* and *stolonifier*) were inoculated. In the case of the *Aspergillus* strain, there was found that only the fractions CF_2_, CF_3_, MF_2_, and MF_3_ showed more than 50% of FGI, being this strain more aggressive than *Rhizopus*.

## 3. Discussion

The EOs selected for this study contained one or two active compounds. This differs from other studies, which is likely due to geographical location, a plant′s adaptive metabolism, harvest time, and extraction conditions [13,14,15,16]. These results are similar to those found by Laribi et al. [15], in which (−)-carvone (76.37 ± 4.73%) and D-limonene (19.52 ± 3.31%) were found to be the main component of Tunisian CEO. However, other studies showed different results. For example, German and Sweden CEO present high yields of limonene (about 70%) followed by carvone [15,17]. Moreover, Egyptian CEO present the dominance of (−)-carvone (48.70%) followed by limonene (24.20%) [18].

The goal was to fractionate the raw oils to obtain their free-terpene hydrocarbon fractions. In fact, the instability of terpene hydrocarbons to heat and light seems to be overshadowing the potential benefits of various essential oils. Thus, it is common industrial practice to remove some or all of the terpene hydrocarbons to concentrate the oxygenate terpenes in the EO. This important operation is referred to as the deterpenation process and obtained fractions are referred to as folded, terpeneless, or deterpenated oils [19,20,21].

Therefore, it was possible to test the bioactivity of the obtained terpenless EO regarding their chemical composition. The quantitative and qualitative differences of the EOs fractions were evident and the antifungal capacity varied according to the distribution of the components of each fraction.

The GC-MS results showed the increase of the proportion of oxygenated compounds in some EO fractions: CF_2_ (79.21%), CF_3_ (98.56%), MF_2_ (85.06%), and MF_3_ (88.86%). Therefore, these were considered as the free-terpene hydrocarbons fractions. With regards to the raw EOs, the CEO could be considered as rich oxygenated but the MEO is moderately oxygenated. The remaining fractions CF_1_ and MF_1_ presented very poor oxygenated compounds but high amount of monoterpene hydrocarbons (79.06% and 87.92%, respectively). These results were similar to those found in previous studies [14,21,22]. Dissimilar results were reported by Laribi et al. (2010) [23], where the terpinene-4-ol chemotype was presented in the *cis*-sabinene hydrate or in combination with *p*-cymene.

The obtaining of high purity (−)-carvone 97.15 ± 5.97% could be considered as an important finding given it has been the subject of extensive research [14]. This fact is of great economic interest due to carvone’s application as a fragrance, flavor, potato sprouting inhibitor, and antimicrobial agent. It also has applications in the medical field [23].

Regarding the fungal strains used in this study, they were chosen because they commonly appear as food spoilage agents. Specifically, the genders *Rhizopus* and *Aspergillus* are the most common agents that deteriorate food [14,16]. Considering that our antifungal results showed that the clearest inhibitory effect occurs in the free-terpene hydrocarbons fractions CF_3_, MF_2_, and MF_3_ with values above 97% against all the strains. This effect could be attributed to the oxygenated terpenes compounds, which represent 98.56% (CF_3_), 85.06% (MF_2_), and 88.86% (MF_3_) of their chemical composition. The results reveal that among all terpene compounds present in the EOs, the oxygenated are those that present higher antifungal activity. Other studies relate oxygenated compounds to bioactivity [3,10,23]. The physical nature of the rich oxygenated EOs, such as their low molecular weights combined with a lipophilic character, allow them to penetrate the cell membrane more quickly than other substances [8,9]. Conversely, the rich monoterpene hydrocarbons fractions CF_1_ (80.49%) and MF_1_ (87.92%) in which the principal compounds were γ-terpinene, *p*-cymene, α-pinene, β-pinene, and limonene exhibited moderate antifungal activities. These results shown that the monoterpenes hydrocarbons possess a limited antifungal potential, which is a conclusion also discussed by other authors [8,23].

According to Morcia et al. 2012 [11], the general classification of the antifungal activity of the compounds present in the EOs from the higher to the lower, show the following order: phenols > aldehydes > ketones > alcohols > esters > hydrocarbons. Therefore, the correlation between the higher antifungal activity and the fractions MF_2_ and MF_3_ is evident, with the presence of a high percentage of alcohols related compounds, such as terpin-4-ol (54.39% and 48.60%, respectively). This is in agreement with other studies, which found that terpinen-4-ol were the primary active components of the EOs [3,11].

In general, the bioactivities of any EO are decided by either one or two of its main components [24]. Thus, the high percentage of (−)-carvone in CEO (74.25%) and its fractions CF_2_ (78.21%) and CF_3_ (97.15%) could be responsible for the high antifungal effect. These results cohered with published data that reported how carvone has been shown to be a biologically active component of Tunisian caraway essential oils. It can be a potato sprouting inhibitor [25,26], antimicrobial agent [18,26], and applied in the medical field [23].

In another set of results, inhibitions against *R. oryzae* and *R. stolonifier* for both CEO (83.68 and 84.29%) and MEO (85.84 and 53.40%) could be considerable in comparison with treated fractions CF_2_ (88.49 and 87.93%), CF_3_ (100% both), MF_2_ (99.84 and 100%), and MF_3_ (97.02 and 98.71%). This might be related to the quantity of the main component of each essential oil CEO (carvone: 74.25%) and MEO (terpine-4-ol: 27.32%). These two last cited terpenes known to have pronounced antimicrobial activities, but their actions depended on the synergistic effect with other constituents of the essential oils [11,18,27]. However, the same conclusion cannot be made for the CEO and MEO inhibition against *A. penicillioides*. The above findings show that the *Rhizopus* and *Aspergillus* fungus families express different behaviors towards the same tested essential oils. Moleyar and Narasimham demonstrated that susceptibility or resistance of a fungus to the antifungal activity of an essential oil depends on the capacity of the fungus to detoxify their main compounds [28]. Thus, *Aspergillus* and *Rhizopus* indicated dissimilar detoxification behavior toward terpinene-4-ol and carvone. This could explain different measured inhibition values. Many parameters affect the relative capacities of the two fungus to detoxify the compounds as enzyme content, microbial transformation, or degradation of antifungal compounds and the mechanism involved [28,29].

## 4. Materials and Methods

### 4.1. Solvents and Reagents

The following reagents were used: Ethyl acetate (high performance liquid chromatography (HPLC) grade, chemical abstracts service (CAS) N° A4A-78-6, purity 99.98%, Fisher chemical Scientific, Loughborough, UK); Lactophenol blue solution (N° 61335, Panreac, Barcelona, Spain); potato dextrose agar (PDA, CAS N° 01-483-500, Eur; Pharm., Scharlab Microbiology, Barcelona, Spain); and *Aspergillus niger* (*A. niger*, Tiegh MB284309, CBS-KNAW, Utrecht, The Netherlands).

### 4.2. Essential Oils Extraction

In July 2015, fresh plant material, including twigs, leaves, and flowers of marjoram (*Origanum majorana* L.) were collected in Sidi Bouzid, Tunisia. In July 2017, seeds of caraway *(Carum carvi* L.) were collected from cultivated plants in the region of Nabeul, Tunisia. These species were verified by the senior taxonomist, Dr. Ridha El Mokni (Laboratory of Botany and Plant Ecology, University of Carthage, Carthage, Tunisia), who confirmed the taxonomic identification of the plant material.

The plant material was deposited in clean and dry plastic bags and processed immediately for the isolation of essential oils. The extraction method was done with a Clevenger apparatus (Fisher Scientific, Toledo, Spain) according to the description of British Pharmacopoeia (1980). From each plant, 400 g were extracted with 3 L distilled water for 3 h at 373.15 K. The collected essential oils were dried under anhydrous sulfate and then stored at 4 °C for further analysis. Hydrodistillation of *Origanum majorana* L. essential oil (MEO) and *Carum carvi* L. essential oil (CEO) yielded 1.7% and 2.8% (*w/w*), respectively. Each extraction was performed at least three times and the standard deviation of the procedure was recorded within the results. The collected data were statistically analyzed according to the normality and homogeneity values of variances by a multiple comparison procedure analysis of variance (ANOVA).

### 4.3. Deterpenation Process

The deterpenation process was carried out according to the method described by Ben Salha et al. (2017) [10]. CEO and MEO were subjected to the vacuum distillation process under different boiling temperatures. Each EO was placed in a boiling flask (capacity 50 mL) and the separation level of the mixture compounds was checked according to the boiling temperature at the top of the column. The constant value was kept at 10 kPa. The distillation was performed until the volume in the flask was totally reduced. The collected samples were analyzed using the gas chromatography–mass spectrometry (GC-MS) technique.

### 4.4. Identification and Quantification of EOs and Their Fractions

The equipment used for the chemical identification was a gas chromatograph coupled with an Agilent 5975C mass spectrometry detector (Agilent Technologies, Inc., Santa Clara, CA, USA) equipped with ionization voltage in the EI-mode (70 eV 166). The oven temperature program was 60 °C rising at 2 °C/min to a final temperature of 280 °C. The functional conditions were: let mode: 10:1; flow rate: 0.7 mL/min; carrier gas: N_2_; and injection volume: 10 μL of essential oil dissolved in 1 mL of ethyl acetate (HPLC grade, CAS N° A4A-78-6, purity 99.98%, Fisher chemical Scientific, Loughborough, UK). The compounds were identified by their retention times [30] using the NIST Mass Spectral Search Program (Mass Spectral Library version 2.2, 2017, National Institute of Standards and Technology (NIST), Gaithersburg, MD, USA). The quantification of each peak was performed by the mass reported. The results were expressed as a percentage (*w/w*).

### 4.5. Isolation of Food Spoilage Fungi

The microorganisms used in this study were isolated from pumpkins, peaches, and prunes, which are available in any market. The isolation method of fungi was previously described by Toma and Abdulla 2013 and Romero et al. 2005 with some modifications [31,32]. The sub-culturing of the fungal colony was carried out using a sterile fresh medium of potato dextrose agar (PDA CAS N° 01-483-500, Eur; Pharm., Scharlab Microbiology, Barcelona, Spain) and incubating at 27 °C until fungal proliferation occurred on the medium surface. The isolation in the culture medium was performed by three replicates.

### 4.6. Identification of the Isolated Fungi

The identification was made according to taxonomic keys based on “habit characters” [16]. The isolated fungi were subjected to morphological and optical studies using microscopic techniques, as well as using an imaging processing and quantification system (Cellometer^®^ Mini and automatic cell counter, Nexcelom Bioscience LLC., Lawrence, MA, USA). The parameters taken into account in the identification were the growth rate, colony diameter, texture, color, reproductive structures, reverse pigmentation, among others [33,34].

### 4.7. Antifungal Activity

The evaluation of the antifungal activity of MEO, CEO, and their fractions was performed by the micro-atmosphere test, according to the protocol described by Ben Salha et al. [10]. Petri dishes (6 cm diameter) containing a PDA medium were aseptically inoculated with 40 μL of each fungal suspension (10^6^ spores/mL). Sterile paper discs (Whatman filter paper grade 1) were impregnated with 5 µL of each EO sample, placed on the agar media, and incubated at 27 ± 1.5 °C. After seven days, the disc was extracted, washed, vortexed, and stained with Lactophenol blue solution to count the spore concentration on the discs with a Cellometer^®^ Mini automated cell counter (Nexcelom Bioscience LLC., Lawrence, MA, USA). Each experiment was carried out twice. The fungi growth inhibition (FGI) was calculated as the concentration of spores per milliliter according to the following Equation (1):

(1) $$\mathrm{FGI}\;\left(\%\right)=\frac{C_{c}-C_{i}}{C_{c}}\times 100$$

C_c_ is the average concentration in the control sample and C_i_ is the average concentration in the treated one.

### 4.8. Statistical Analysis

The collected data were statistically analyzed according to the normality and homogeneity values′ of variances by a multiple comparison procedure analysis of variance (ANOVA). The Bonferroni Significant Difference (BSD) and the Tukey test were applied after rejecting the null hypothesis. The software used for this statistical analysis was Origin 2017 (OriginLab Corporation, Northampton, MA, USA).

## 5. Conclusions

Vacuum distillation is an effective method to isolate oxygenated terpenes from carum and origanum EOs. This method found values of 79.21%, 98.56%, 85.06%, and 88.86% in the fractions CF_2_, CF_3_, MF_2_, and MF_3_, respectively. Furthermore, it was found that these fractions presented the most substantial fungal growth inhibition against the tested strains and thus their bioactivity was directly connected with the high content of oxygenated compounds (alcohol: terpin-4-ol, and ketone: (−)-carvone). According to these results, these fractions rich in oxygenated compounds can be used in formulations as natural additives against food spoilage, as they present high extraction yields that are interesting for industrial applications.

## Figures and Tables

**Table 1:** Chemical composition of essential oils and fractions identified by the gas chromatography–mass spectrometry (GC-MS).

Compounds	*m/z*	RT	CEO	CF1	CF2	CF3	MEO	MF1	MF2	MF3
			(%)	(%)	(%)	(%)	(%)	(%)	(%)	(%)
β-terpinen	93/77/79	5.72	0.03 ± 0.02	0.08 ± 0.03	-	-	-	-	-	-
β-myrcen	93/69/41	5.91	0.18 ± 0.02	1.54 ± 0.25	1.15 ± 0.19	-	-	-	-	-
octanal	41/43/57	6.08	0.03 ± 0.01	0.55 ± 0.08	0.03 ± 0.00	-	-	-	-	-
2-tujene	93/9177	6.21	-	-	-	-	2.24 ± 0.42	4.12 ± 1.04	-	-
α-pinene	93/91/92	6.39	0.05 ± 0.02	0.14 ± 0.02	0.05 ± 0.01	0.49 ± 0.08	0.87 ± 0.16	1.61 ± 0.25	-	-
D-limonene	68/93/67	6.55	22.22 ± 2.13	77.32 ± 4.01	18.31 ± 1.96	0.07 ± 0.01	-	-	-	-
camphene	93/121/79	6.79	-	-	-	-	0.08 ± 0.05	-	-	-
4(10)-Thujene	41/94/69	7.52	-	-	-	-	5.53 ± 0.41	11.17 ± 1.03	-	-
β-linalool	71/93/43	7.54	0.05 ± 0.01	0.08 ± 0.02	0.09 ± 0.02	0.03 ± 0.00	-	-	-	-
β-pinene	28/32/93	7.60	-	-	-	-	0.46 ± 0.07	0.90 ± 0.09	-	-
1*R*,4*R*-*p*-mentha-2,8-dienol	109/79/94	7.97	0.20 ± 0.02	0.15 ± 0.01	0.30 ± 0.05	0.15 ± 0.03	-	-	-	-
myrcene	28/93/41	8.05	-	-	-	-	1.62 ± 0.09	2.77 ± 0.88	-	-
*cis*-*p*-Mentha-2,8-dien-1-ol	134/109/43	8.22	0.34 ± 0.06	0.06 ± 0.00	0.44 ± 0.06	0.14 ± 0.04	-	-	-	-
limonene oxide	43/94/67	8.29	0.07 ± 0.00	-	0.39 ± 0.10	0.03 ± 0.00	-	-	-	-
α-phellandrene	93/91/77	8.44	-	-	-	-	0.58 ± 0.11	0.95 ± 0.14	-	-
2-nonenal	55/28/41	8.64	0.04 ± 0.00	-	0.07 ± 0.02	-	-	-	-	-
camphenone	93/108/91	8.85	0.05 ± 0.00	-	0.07 ± 0.01	0.04 ± 0.01	-	-	-	-
α-terpinene	121/93/136	8.87	-	-	-	-	11.08 ± 0.88	21.15 ± 4.52	1.48 ± 0.00	0.18 ± 0.00
*p*-cymene	119/28/134	9.08	-	-	-	-	2.76 ± 0.54	5.20 ± 0.48	0.64 ± 0.08	0.15 ± 0.04
2-isopropyl-5-methyl-4-hexanal	69/84/41	9.11	0.33 ± 0.09	-	0.47 ± 0.04	0.10 ± 0.02	-	-	-	-
β-phellandrene	93/77/91	9.23	-	-	-	-	4.36 ± 0.25	7.81 ± 0.78	0.87 ± 0.06	0.51 ± 0.07
γ-terpinene	93/91/136	10.27	-	-	-	-	15.73 ± 1.20	27.53 ± 2.45	7.08 ± 1.02	-
carveol	84/109/134	10.33	1.55 ± 0.13	-	0.65 ± 0.07	0.32 ± 0.02	-	-	-	-
trans-sabinene hydrate		10.47	-	-	-	-	1.58 ± 0.24	1.30 ± 0.07	2.84 ± 0.17	0.44 ± 0.07
7dihydrocarveol	93/107/121	10.49	0.22 ± 0.02	-	0.17 ± 0.03	0.67 ± 0.09	-	-	-	-
(−)-Carvone	82/108/93	11.07	74.25 ± 4.25	20.18 ± 1.63	78.21 ± 3.69	97.15 ± 5.97	-	-	-	-
α-Terpinolene		11.20	-	-	-	-	3.82 ± 0.56	-	2.03 ± 1.11	3.43 ± 0.89
*cis*-sabinene hydrate		11.53	-	-	-	-	4.52 ± 0.76	1.88 ± 0.30	10.00 ± 1.56	5.79 ± 0.78
linalool	71/28/93	11.60	-	-	-	-	1.16 ± 0.09	0.39 ± 0.01	1.82 ± 0.77	0.67 ± 0.22
perilla aldéhyde	135/77/93	11.82	0.21 ± 0.02	-	0.22 ± 0.10	0.67 ± 0.14	-	-	-	-
*trans*-*p*-menth-2-enol		12.30	-	-	-	-	2.05 ± 0.02	-	3.13 ± 0.92	1.90 ± 0.41
*cis*-β-Terpineol	28/43/93	12.92	-	-	-	-	1.36 ± 0.33	0.52 ± 0.09	1.86 ± 0.20	2.76 ± 0.38
endo-borneol		13.85	-	-	-	-	0.23 ± 0.04	-	-	0.46 ± 0.05
thymol	135/150/91	14.23	-	-	-	-	-	-	-	0.30 ± 0.06
terpine-4-ol	71/111/93	14.35	-	-	-	-	27.32 ± 2.21	7.30 ± 1.22	54.39 ± 3.25	48.60 ± 4.87
β-fenchyl alcohol		14.40	-	-	-	-	-	-	9.63 ± 1.54	23.84 ± 2.39
*p*-cymen-8-ol		14.50	-	-	-	-	0.08 ± 0.00	-	-	-
*trans*-piperitol		14.81	-	-	-	-	0.64 ± 0.04	-	0.57 ± 0.07	2.61 ± 0.37
*cis*-piperitol	28/32/18	15.17	-	-	-	-	0.73 ± 0.09	-	0.82 ± 0.04	1.79 ± 0.11
carvacrol	135/150/91	17.89	-	-	-	-	0.21 ± 0.00	-	-	-
caryophyllene	133/93/91	20.78	-	-	-	-	0.83 ± 0.07	-	0.80 ± 0.04	2.70 ± 0.44
aromandendrene		21.23	-	-	-	-	0.05 ± 0.00	-	-	0.33 ± 0.10
α-humulene		21.55	-	-	-	-	0.05 ± 0.01	-	-	-
(+)-Bicyclogermacrene	28/32/18	22.50	-	-	-	-	0.65 ± 0.20	-	-	1.68 ± 0.17
spathulenol	43/41/205	24.18	-	-	-	-	0.24 ± 0.04	-	-	0.20 ± 0.02
caryophylleneoxide	79/43/93	24.30	-	-	-	-	0.15 ± 0.00	-	-	-
linalylacetate	93/28/43	16.60	-	-	-	-	0.56 ± 0.10	-	0.65 ± 0.09	1.23 ± 0.22
Isobornyl acetate	28/32/95	17.41	-	-	-	-	0.26 ± 0.02	-		0.41 ± 0.03
α-terpenylpropionate		17.61	-	-	-	-	-	-	1.39 ± 0.21	-
4-Terpinenyl acetate	93/121/136	17.81	-	-	-	-	1.03 ± 0.18	-	-	-
Mass (g)			20.12	4.1	5.62	10.40	22.00	8.88	10.13	0.99
Boiling temperature (at 10 mmHg)			-	65–75	80–90	110	-	52–54	70–72	84–86
Total terpenes hydrocarbons (%)			22.48	79.06	19.47	0.56	50.70	87.92	12.90	8.98
Total oxygenated terpenes (%)			77.43	21.62	79.21	98.56	47.36	12.05	85.06	88.86

**Table 2:** Fungal growth inhibition (FGI %) of CEO and MEO against *R. oryzae, R. stolonifier,* and *A. penicillioides.*

Samples	CEO	CF_1_	CF_2_	CF_3_	MEO	MF_1_	MF_2_	MF_3_
% FGI *R. oryzae*	83.68 ± 1.75	41.51 * ± 5.95	88.49 ± 0.12	100.00 ± 0.00	85.84 ± 1.84	16.41 * ± 1.04	99.84 ± 0.22	97.02 ± 4.21
% FGI *R. stolonifier*	84.29 ± 2.56	43.69 * ± 1.38	87.93 ± 1.96	100.00 ± 0.00	53.40 * ± 0.58	12.85 * ± 3.58	100.00 ± 0.00	98.71 ± 1.81
% FGI *A. penicillioides*	40.15 ± 4.68	19.92 ± 0.85	84.01 ± 12.62	100.00 ± 0.00	29.95 ± 3.79	11.53 ± 2.17	100.00 ± 0.00	98.29 ± 2.41

The population means are significantly different according to analysis of variance (ANOVA) one-way test at the 0.01 level. * The difference of the means is significant at the 0.01 level when comparing with all samples, according to Bonferroni and Tukey tests.
